# Addressing the needs of infants with prenatal substance exposure: implementation of CAPTA and CARA legislation in an urban hospital setting

**DOI:** 10.3389/fpubh.2025.1549228

**Published:** 2025-03-26

**Authors:** Anita Jose, Scott Wetzler, Julie R. Kaplan, Angel Mendoza, Jacqueline Martin, Wenzhu Mowrey

**Affiliations:** ^1^Department of Psychiatry and Behavioral Sciences, Montefiore Medical Center, Bronx, NY, United States; ^2^Department of Obstetrics and Gynecology and Women’s Health, Montefiore Medical Center, Bronx, NY, United States; ^3^New York City Administration for Children’s Services, New York, NY, United States; ^4^Department of Biostatistics, Albert Einstein College of Medicine, Bronx, NY, United States

**Keywords:** CAPTA notification, child welfare, infants with prenatal substance exposure, plans of safe care, substance use disorders

## Abstract

**Introduction:**

In recent years, federal legislation, including the Child Abuse Prevention and Treatment Act (CAPTA) and the Comprehensive Addiction and Recovery Act (CARA), have focused on the needs of infants with prenatal substance exposure (IPSE). This manuscript provides data from the implementation of this legislation in a large urban hospital setting.

**Method:**

This paper provides data on 403 mothers and infants at risk of IPSE, who were recruited from a large NYC hospital system through case record review and verbal screening. Participants provided self-report data maternal substance use, mental health, and trauma; administrative data were also obtained from the electronic medical record and the state child welfare database.

**Results:**

In this sample, 10.7% of at-risk mothers met DSM5 criteria for active substance use disorder (SUD), while an additional 36.8% met criteria for SUD in remission. Moreover, 7.5% of mothers met state eligibility for a Plan of Safe Care (POSC). Mothers were more likely to report mental health symptoms than active substance use, with 34.1% reporting moderate-to-severe depressive symptoms and 27.8% reporting moderate-to-severe anxiety. Mothers with active SUDs were more likely to experience the most severe mental health symptomatology. As for infants, 1.6% met the threshold required for notification under CAPTA at birth. During the 6-months after birth, 4.2% infants were involved in an indicated report of child maltreatment, and 1.0% were placed in out-of-home care.

**Discussion:**

These findings suggest that, although CARA and CAPTA legislation were intended to address a high prevalence of IPSE, some of the concerns driving this legislation may be overstated as, based on our data, the actual number of affected infants is relatively small, even in an at-risk population.

## 1 Introduction

In the United States, more than one in ten (12.3%) children under the age of 18 have experienced living firsthand with parental substance use disorders (SUDs) (1). Parental substance use is associated with higher rates of foster care placement, which is especially concerning as substantiated reports of parental drug and alcohol use have also increased since the early 2000s (2, 3). These data underscore the need to address substance use in families to prevent adverse child safety outcomes. Given the differential impact to younger children, it’s possible that addressing this risk factor early (even prenatally) can serve as a meaningful method of prevention.

Nationally representative epidemiological studies have found that although pregnant people were significantly less likely to use substances than those who were not pregnant, a number of pregnant individuals still reported using substances in the prior month, most frequently alcohol and cannabis (4, 5). Beyond the fact that almost all drugs are known to cross the placenta and have an impact on development of the fetus, prenatal substance exposure has been associated with a greater risk of stillbirth, or Sudden Unexpected Death in Infancy (SUDI), and other medical issues (e.g., problems with fetal growth, congenital abnormalities, neonatal withdrawal symptoms, neurobehavioral issues) (6, 7) Consequences of prenatal substance exposure can be observed in the longer term as well, including behavioral and cognitive challenges, and delays in language development (7).

Pregnancy may be a crucial time in which to address substance use. Indeed, many parents-to-be are highly motivated to address health and lifestyle issues to support the baby’s well-being during the transition to parenthood. For instance, one study found that, upon learning they were pregnant, many women completely ceased their substance use, while others engaged in harm reduction by reducing their use (8). Though partly dependent on state laws and regulations regarding substance use during pregnancy, prenatal intervention may mitigate adverse infant outcomes, and potentially decrease child welfare involvement.

Given the impact of prenatal and perinatal substance use, the U.S. government has enacted legislation to undertake the issue on the national level. The Child Abuse Prevention and Treatment Act (CAPTA) was first passed in 1974. Early iterations did not address the needs of infants exposed in utero to drugs and alcohol, but starting in the early 2000s, language specific to prenatal substance exposure began to be included in the legislation (9, 10). As of 2018, CAPTA requires that states provide the federal government a tally of the number of “substance affected” newborns, though no identifiable patient health information (PHI) is used, and there is no associated clinical reporting requirement. Perhaps intentionally, federal language operationalizing what it means to be “affected by” substance is vague (although it must include infants with Fetal Alcohol Spectrum Disorder/FASD, withdrawal symptoms including Neonatal Abstinence Syndrome/NAS, or both). CAPTA also requires that Plans of Safe Care (POSCs) be completed to address the needs of these infants.

The Comprehensive Addiction and Recovery Act (CARA) was signed into law in 2016 during the start of the US opioid epidemic, with a focus on enhancing substance use related services (10). CARA modified CAPTA by requiring POSCs to address the needs of both infant *and* caregivers, and to address the needs of infants impacted by illegal *and* legal substances. States have discretion in how to implement and apply POSCs, although the ultimate responsibility for POSC development lies with the state child welfare agency (11).

As the federal language is relatively broad, states have latitude in how to interpret these requirements. A recent review shows that, of all states plus Washington, D.C., only two were fully compliant with CAPTA requirements (10). The two states in full compliance with these requirements (Delaware and North Carolina) have published data. In Delaware, 93.8% of infants identified as having been born with prenatal substance exposure had a POSC (12). Most infants (67.2%) were exposed to one substance (with 74.4% of those exposed to cannabis only), and 97.9% of infants with any prenatal substance exposure were referred for further child welfare screening (while 11.3% were placed in out-of-home care, and 0.8% experienced significant physical injury or fatality). The placement rate in Delaware seems quite high, especially when the major substance of exposure in the sample seems to have been cannabis. Data from North Carolina similarly demonstrated that, in a sample comprised only of children with POSCs referred to child protective services, toxicology screens were positive for about 90% of infants, with the vast majority testing positive for cannabis only (70.3%); 4.6% of infants were placed in out-of-home care (13). Although these data reflect only cases where child welfare services were notified of IPSE, this pattern suggests that in these two states, many newborns were exposed to substances prenatally (mostly cannabis), and POSCs were used to track parent and infant service needs. It also seemed that psychosocial correlates, as well as involvement with child protection, were associated with prenatal substance exposure. As CAPTA and CARA legislation have been implemented in different ways in different states, it is incumbent upon providers to be aware of the ethical and legal complexities involved in addressing substance use during pregnancy on the patient, provider, and systemic levels, and in addressing the clinical needs of pregnant people (14).

There are many barriers to addressing prenatal substance use. For instance, on the patient level, individuals using substances during pregnancy may feel ashamed or may be concerned about legal or child welfare repercussions; not only does this preclude the individual’s engagement in treatment to address substance use, but it also causes delays or absences in prenatal care. Even if such patients attend appointments regularly, they may be reluctant disclose substance use to their provider.

In addition to personal stigma, people residing in many parts of the U.S. may indeed face legal repercussions. Twenty-four states, as well as D.C., consider substance use during pregnancy to fall under civil child-welfare statutes, and a similar number require healthcare providers to report suspected prenatal drug use (15). This means that parents who use substances during pregnancy may lose parental custody and/or have their parental rights terminated. In other states, substance use can lead to civil commitment. Finally, certain states criminally prosecute pregnant people who use drugs (16).

Importantly, both the American College of Obstetricians and Gynecologists (ACOG) and the American Academy of Pediatrics (AAP), as well as many other clinical and public health entities indicate that such criminal justice interventions have not been shown to be effective and may instead increase barriers to care for this high-risk population (17, 18).

Several potential barriers also exist for providers. For instance, in some areas, there are geographic barriers to accessing appropriate treatment, including treatment to address substance use during pregnancy. Another concern is related to screening. ACOG recommends universal verbal screening using a validated measure for all pregnant people. However, providers may have limited time to screen patients and may not know which screening tools to use. They may also believe that urine toxicology screens may identify more substance use than patient self-report when in actuality, the panel of substances being tested and the recency and intensity of substance use may impact results. This issue is particularly salient since research shows a disproportionate impact of urine toxicology on racial minority populations (19, 20).

Social attitudes have changed rapidly over the past several years related to certain substances (i.e., marijuana legalization) and there may be limited empirical evidence about short- and long-term impacts on mother and infant (especially compared to research on substances like alcohol and tobacco, which have been legal for a very long time). At the same time, state-level reporting requirements risk conceptualizing substance use as a moral issue, rather than viewing SUDs as chronic, relapsing, bio-behavioral conditions. In such a confusing context, providers’ personal attitudes on substance use during pregnancy may impact their clinical decisions.

Finally, providers may find themselves challenged as they navigate concerns about patient confidentiality. For instance, the Confidentiality of Substance Use Disorder Patient Records, 42 Code of Federal Regulations (“42 CFR Part 2”) requires express consent of the patient when disclosing any substance use information. However, providers may not be aware that when there is a related safety concern (i.e., child maltreatment), 42 CFR Part 2 restrictions do not apply to these mandated reporting disclosures.

Systemic barriers (such as limited cross-systems collaboration between various types of providers, and different philosophies across different provider systems) should also be considered for their role in preventing access to prenatal substance use care. Obstetricians, neonatologists, SUD treatment providers, and child welfare providers (all of whom may be involved in a case where substance use occurred prenatally or perinatally) may each see the “patient” and the goals of treatment differently. Moreover, the person being treated by the provider may naturally be viewed as the provider’s priority and even an object of some professional loyalty.

In another example of a systemic barrier, child welfare systems have been viewed as part of institutionalized racial and social prejudice for decades. For instance, Perritt writes, “Most mandated reporters, such as physicians, have been taught that the child welfare system is an unbiased legal system that ensures children’s safety and well-being. In reality, indications for reporting, reasons for removal of children, and subsequent monitoring and surveillance are racially biased, subjective, and paternalistic” (21). This impactful opinion piece in the New England Journal of Medicine considered how to prevent criminalization of mothers who experience the clinical problem of substance use, while exploring how clinical information may be misused by governmental systems. Race and the criminalization of substance use during pregnancy are indeed important and related systemic concerns, as Perritt describes. In fact, there is a disproportional impact of SUD reporting requirements on Black communities, which can be tied into the concept of “misogynoir” – the intersection between sexism and racism (22). Moreover, once involved in the child welfare system, many Black and Latinx parents feel judged and intimated by workers and stigmatized within their communities (23). Taken together, institutionalized racism may impact which families are referred to the child welfare system and may then adversely affect the experiences of the families receiving these services.

Accurately assessing substance use is not only a challenge on the patient and provider levels, this is also a systemic difficulty. Even when providers make assessments, and patients consent, there are challenges with the measures themselves: verbal screenings may be unreliable due to under-reporting, laboratory measures (including urine toxicology, hair, or meconium testing) may be applied disproportionately on certain populations, and clinical measures (such as evaluating infant withdrawal symptoms at birth) only capture significant, recent use of specific types of substances (such as opioids).

Prenatal substance use is clearly a significant concern, and one for which there are many patient, provider, and systemic barriers. However, intervention during pregnancy may be particularly beneficial, not only because of the unique motivation many pregnant individuals feel during the transition to parenthood, but also because early identification of substance use problems during pregnancy, paired with individualized service planning required by CAPTA and CARA (i.e., POSCs) may achieve the goal of helping families, perhaps even decreasing the risk of child welfare involvement. However, the topic of prenatal substance engenders strong political opinions on a broader scale, which is why different states have treated this issue in different ways. Although New York, where this research was conducted, does not criminalize prenatal substance exposure directly, criminal charges may be brought for allegations related to child abuse more broadly; regardless, we are still faced with clinical and procedural challenges when implementing this legislation (24).

New York State, specifically New York City, has a unique perspective into this issue, as a large, urban area with a high concentration of health care and SUD treatment facilities and a robust child welfare system. This study explores the extent of the problem and the issues surrounding CAPTA implementation in a large, urban medical center. In the Bronx, there were 17,749 births per year (3-year average) (25), and approximately one quarter of these babies were delivered by the academic medical center where this study was conducted (26), demonstrating that this healthcare system captures a significant proportion of Bronx deliveries.

Our aim is to determine the impact of substance use, and concomitant symptoms (including mental health symptoms, and child welfare involvement), on pregnant people and their infants. This is particularly timely because of the recent CAPTA/CARA legislation that suggests maternal substance use during pregnancy or delivery has adverse impacts. Specifically, we hypothesize that:

1. Given the prevalence of substance use in the community, we anticipate that a subgroup of this sample will be at risk of active substance use during pregnancy.
2. This sample will exhibit clinically elevated rates of depression, trauma, and anxiety, as these symptoms often co-occur with both pregnancy and substance use.
3. Given that federal legislation has identified substance use as a problem, a meaningful number of these individuals will meet criteria for Plans of Safe Care, CAPTA notification, and/or child welfare reports.

## 2 Materials and methods

### 2.1 Participants

This study was approved by the Albert Einstein College of Medicine Institutional Review Board (IRB). Additional approval for the inclusion for administrative child welfare data in this project was obtained from the NYC Administration for Children’s Services (NYC ACS) Research Review Board and the NYS Office of Children & Family Services (NYS OCFS) Review Board.

Adult (18+) participants were recruited through a large urban Department of Obstetrics & Gynecology due to a pregnant (16+ weeks pregnant) or postpartum status (up to 12 weeks) and being identified as at high risk of substance misuse through the Substance Use Risk Profile- Pregnancy (SURP (27)), a positive urine toxicology, or a clinician referral.

Participants were recruited from a large healthcare system, which delivered about one fourth of all babies born in the Bronx during the study period. During the recruitment period (from 2018 to 2022), this included a universe of 24,178 adult patients that received prenatal care or delivered their infants within this system. These potentially eligible charts were randomized, then reviewed sequentially by a study recruiter until the total study enrollment goal was met. Over the course of the study, the recruiter screened 18,284 charts (approximately 76% of the total) to determine possible risk of substance use (defined as: a substance use disorder diagnosis, a positive toxicology during or prior to pregnancy for the birthing parent, a positive toxicology at any point for the infant, or identification of any kind of substance use on the patient’s “problem list” by any provider) and found that 837 charts suggested possible risk of substance use. If the medical record indicated a positive toxicology during pregnancy or at delivery (or if the patient was directly referred by a clinician), the participant was eligible and their interest in participating was ascertained by the recruiter. For all other potential participants, the study recruiter conducted the SURP-P screening to determine eligibility. Of this at-risk group, all 837 individuals were contacted, 65% expressed interest, and 403 (approximately 48% of all those identified as at-risk by the initial chart review) were enrolled into the study.

Eligible and interested participants were invited to meet with study staff to complete the Informed Consent process, completing a battery of self-report questionnaires (and consent to disclose medical and child welfare administrative data). Participants were able to participate in person or remotely (by video conference or telephone) and were able to receive translation services if requested (including receiving consent forms and questionnaires in English or Spanish). All participants received renumeration for their time. Data were collected from 403 individuals who were recruited between 2018 and 2022 (all respondents identified as female).

### 2.2 Measures

#### 2.2.1 Screening measures

##### 2.2.1.1 Substance use risk profile- pregnancy (SURP)

This 3-item screening for risk of substance use during pregnancy was found to have good sensitivity and specificity in a sample of pregnant women who presented for obstetric care in a hospital setting (27). Participants whose screens suggested high risk (2+ positive responses) were invited to participate in the study.

##### 2.2.1.2 Urine toxicology

Participants were referred due to urine toxicology screenings that were conducted for clinical reasons; this means that different panels may have been used depending on the provider’s needs. However, panels generally included testing for: cannabis, opioids, oxycodone, methadone, benzodiazepine, cocaine, amphetamine, buprenorphine, and/or barbiturates. Participants were enrolled if their records indicated a positive urine toxiciology during pregnancy or at delivery.

#### 2.2.2 Maternal substance use

##### 2.2.2.1 Addiction severity index (ASI)

This assessment measures alcohol and substance use and related functioning (28). Given this project’s focus, only the drug and alcohol scales were utilized. An alcohol use score of 0.20 or higher, or a drug score of 0.15 or higher, indicates high use for women. Internal consistency for this measure has a wide range, from 0.44 to 0.89 (29).

##### 2.2.2.2 Diagnostic interview

Participants completed a structured interview with a licensed social worker to determine whether they met DSM5 criteria for current or past SUD.

##### 2.2.2.3 Electronic medical record

The electronic medical record (EMR) was reviewed for any urine toxicology screens (and results) during the 6-months after enrollment. In addition, some participants were referred due to a positive screen, which could have occurred at any period prior to enrollment.

#### 2.2.3 Maternal mental health

##### 2.2.3.1 Beck anxiety inventory (BAI)

This questionnaire measures anxiety in the prior month (30). Scores range from 0 to 63; moderate anxiety is defined as scores between 16 and 25 and severe anxiety is defined as scores between 26 and 63. Originally tested in psychiatric populations, this measure also has high internal consistency in non-clinical populations, though low test–retest reliability suggest it is measuring state-level anxiety, rather than trait anxiety (31).

##### 2.2.3.2 Center for epidemiological studies depression, 12-item short form (CES-D)

This abbreviated version of the Center for Epidemiological Studies – Depression Scale asks about mood in the week prior, with scores ranging from 0 to 36 (32). Moderate depression is defined as a score of 11–14, while severe depression is a score of 15 or higher. The short form used in this study has good internal consistency (0.83–0.92), and has also been used in large scale studies with populations parents (29).

##### 2.2.3.3 Trauma symptoms checklist – 40 (TSC-40)

This measure assesses trauma symptomatology (33). Given the project’s focus, only the Total Symptoms score was analyzed. Among females, a Total Symptoms score of 46 or higher is clinically elevated. Internal consistency is high (0.89–0.91) in large samples of women (29).

#### 2.2.4 Plan of safe care eligibility

The EMR as well as SUD diagnostic information were reviewed to determine whether enrollees may be eligible for Plans of Safe Care (POSCs). In New York State, POSCs are developed for pregnant people who (1) are diagnosed with an SUD, or (2) are receiving medication assisted treatment (MAT) for an SUD, or (3) are under the care of a healthcare provider who has prescribed opioids (34). For the purposes of capturing the population whose substance use would then lead to having a substance-affected infant, we excluded pregnant people who had mild or moderate cannabis use disorder (without other active substance comorbidities) in the numerator, when calculating the proportion of this sample eligible for POSCs.

#### 2.2.5 Substance affected infants

The EMR was reviewed to determine whether infants were identified as *substance affected* (and requiring CAPTA notification) at birth based on New York State requirements, including the following criteria: (1) a diagnosis of Fetal Alcohol Spectrum Disorder (FASD); (2) a diagnosis of Neonatal Abstinence Syndrome (NAS) or Neonatal Opioid Withdrawal Syndrome (NOWS), and/or (3) a positive toxicology plus withdrawal symptoms.

#### 2.2.6 Infant child welfare involvement

Administrative data were provided from the New York State Child Welfare Database about substantiated child abuse or neglect reports, and infant removals/foster care placements, within 6 months of birth.

### 2.3 Statistical analysis

De-identified data were scored and analyzed as summary statistics. Continuous variables were summarized as means and standard deviations while categorical variables were summarized as frequencies and percentages. Maternal substance use, mental health, and trauma data were summarized and evaluated. The number of substance-affected infants and the number of child maltreatment reports and placements within 6 months of birth were tabulated and reported. All analyses were conducted with the statistical software, SAS 9.4 (35).

## 3 Results and discussion

### 3.1 Demographics

Most respondents who were identified (81.6%) were eligible to participate because they scored at “moderate” or “high” risk for substance use during pregnancy using a validated verbal screening measure. However, a smaller proportion were identified after receiving a positive toxicology screen for one or more substances (15.2%) and a few participants were enrolled after a direct clinician referral to the study (3.2%). Of the 61 women identified after a positive toxicology screen, the majority tested positive for cannabis only (65.6%), 9.8% were positive for opioids alone (including oxycodone, methadone and buprenorphine), 1.6% were positive for PCP alone, and the remainder were positive for multiple substances (23.0%). The high rates of cannabis use in this sample are consistent with findings from Delaware and North Carolina described earlier.

The average maternal age was 29.4 years (SD = 5.9 years) at enrollment. As illustrated in Table 1, just over half of mothers identified as Hispanic, similar to Bronx rates (36). Most non-Hispanic mothers in this study identified as Black. Compared to the Bronx, this sample was also more likely to have graduated high school (84.5% vs. 74.5%). Participants were also less likely to be in the civilian labor force than the average Bronx female (39.0% vs. 54.7%), although this is likely due to their pregnancy or postpartum status. In terms of infants, about half (49.3%) were female. While infants were more likely to be identified as Hispanic (60.9%) compared to their mothers, race distribution was broadly like their mothers, with the majority identified as Black (31.3%).

**Table 1:** Maternal demographic characteristics.

Variable	Mother (*n* = 403)
Percent female	100.0%
Race & ethnicity	54.1% Hispanic (any race)
	36.7% Black (non-Hispanic)
	6.7% White (non-Hispanic)
	2.5% Other (non-Hispanic)
Employment status	39.0% Employed
Housing status	7.9% in Shelter or Homeless
Relationship status	46.5% Married or cohabiting with infant’s bio parent
Highest education	84.5% Completed high school or beyond
Annual income	45.5% Earn $0 to $9,999
	14.1% Earn $10,000 to $19,000
	7.0% Earn $19,001 to $24,999
	10.6% Earn $25,000 to $34,999
	11.6% Earn $35,000 to $49,999
	11.3% Earn $50,000 or more

### 3.2 Maternal substance use

Enrolled mothers were invited to complete the ASI and meet with a clinician to determine SUD diagnostic status as part of this study. Some participants’ providers recommended toxicology screens for clinically indicated reasons while they were enrolled in this study; therefore, toxicology data are also available for this subset of participants (Table 2).

**Table 2:** Maternal substance use characteristics.

Variable		Mother
DSM5 diagnosis	Active SUD	10.7%
	Alcohol Use Disorder Only (32.3%)	
	Cannabis Use Disorder Only (58.1%)	
	Other/ Multiple SUDs (9.7%)	
	SUD in Remission (in last 5 years)	36.8%
	Alcohol Use Disorder, Any (68.2%)	
	Cannabis Use Disorder, Any (57.9%)	
	Opioid Use Disorder, Any (7.5%)	
	Stimulant Use Disorder, Any (5.6%)	
	Sedative Use Disorder, Any (1.9%)	
	Hallucinogen Use Disorder, Any (0.9%)	
	No SUD Diagnosis (in last 5 years)	52.6%
ASI score	High Drug Use	0.8%
	High Alcohol Use	0.5%
Positive toxicology screen	Positive: Cannabis only	22.7%
	Positive: Opiates/ Oxycodone only	6.8%
	Positive: Methadone only	9.1%
	Positive: PCP only	2.3%
	Positive: Multiple Substances	2.3%
	Negative: All Substances	56.8%

In this sample, just under 11% of women assessed met diagnostic criteria for a current SUD diagnosis, while slightly more than one third had one or more SUD diagnosis in remission (and no active SUD). The remaining individuals did not meet criteria for any current or past SUD in the last 5 years. Although most people with an active SUD reported Cannabis Use Disorder (58.1%) or Alcohol Use Disorder (32.3%) alone, two individuals met current criteria for both Cannabis and Alcohol Use Disorders together while another participant was diagnosed with active Alcohol, Cannabis, and Opioid Use Disorders. Those in remission were often in remission for alcohol alone (34.9%), cannabis alone (24.8%), or both (27.5%). It is worth highlighting the fact that rates for active Opioid Use Disorder were quite low in this sample, especially given that CARA legislation focused on the impact of opioid use during pregnancy. Rather, respondents were more likely to meet criteria for a disorder related to cannabis and/or alcohol during pregnancy.

In comparison, ASI scores (which measure current use) were particularly low, with only three people meeting the threshold for High Drug Use, and two people meeting the threshold for High Alcohol Use. Upon closer review, part of the contrast between ASI scores and SUD diagnoses may be because SUD diagnostic criteria focus more directly on impact of use (not just frequency, duration, and intensity of use as the ASI does). Since the ASI is a self-report measure with high face validity, client presentation bias can also explain lower scores on this measure. On the other hand, formal SUD diagnosis was determined based on a semi-structured interview with a skilled clinician, which may allow clients to feel more comfortable disclosing sensitive information.

A small group of mothers (*n* = 44) had recorded urine toxicology screens within 6-months after program enrollment. Most (56.8%) of these women tested negative for all substances, while positive toxicology screens were most frequently for cannabis. Only one respondent tested positive for multiple substances (cannabis and opiates). The relatively low rate of toxicology screen delivery is likely because in November 2020, the New York City Commission on Human Rights announced an investigation into several NYC hospitals due to concerns that Black and Latin birthing parents and newborns were disproportionally targeted by urine toxicology screens during pregnancy. In response, many hospitals (including ours) updated their processes around requesting urine toxicology screens.

More broadly, we found that in this high-risk sample, about one-tenth of mothers-to-be were diagnosed with an active SUD, and most of these mothers met criteria only for Cannabis Use Disorder. Moreover, only one respondent met criteria for an active Opioid Use Disorder, which is particularly interesting given that the focus on CAPTA and CARA is on opioid use. Although it is difficult to obtain data on prenatal substance use in the Bronx, a national hospital-based sample of mothers found that at delivery, only 1–2% of mothers had an active SUD (excluding tobacco) (37). In comparison, the rate of active substance use found in the current sample is extremly high.

### 3.3 Maternal mental health and trauma

Mothers were asked to complete validated self-report measures on depression (CES-D), anxiety (BAI) and trauma (TSC-40), and many mothers reported experiencing these symptoms at study enrollment. Specifically, although the average CES-D score for this sample was 8.1 (SD = 8.0), reflecting a non-depressed state, over one third (34.1%) of mothers reported CES-D symptomatology consistent with moderate or severe depression. In terms of the BAI, participant average score was 10.7 (SD = 12.5), reflecting mild anxiety, although over one quarter (27.8%) of mothers reported moderate or severe levels of anxiety. In terms of trauma, the average TSC-40 score for this sample was 23.7 (SD = 18.6), reflecting non-clinical trauma symptomatology. However, 12.6% of participants reported clinically elevated trauma symptoms.

A recent review of studies worldwide suggested the prevalence of postpartum depression is approximately 17%, and the prevalence of anxiety is about 10% postpartum, and 15–20% prenatally (38); the rates in this sample are clearly much higher. Similarly, a meta-analysis suggested that in community samples, the prevalence of PTSD was 3.3% (39). Although the current study measures clinically elevated trauma symptoms rather than PTSD, the level of trauma symptomatology in this sample seems higher than may be expected in community samples.

### 3.4 Plan of safe care eligibility

In New York State, pregnant individuals may be eligible for a POSC if they meet one or more of three criteria: an active SUD diagnosis, receipt of MAT for SUD, or being under the care and supervision of a provider who has prescribed opioids. In this sample, although 10.7% of the sample met criteria for an active SUD, when excluding mild- or moderate- cannabis use disorder without any active substance comorbidities, 6.5% of mothers remained. The medical record identified four other mothers (1.0%) whose records indicated they received MAT, and no mothers who were receiving opioids under the supervision of a healthcare provider for other reasons. In total, 7.5% of the sample would be eligible for POSCs under NYS guidelines.

### 3.5 Infant substance exposure

This study began prior to the official implementation of CAPTA/CARA in New York State, which began in early 2022. However, the medical record was reviewed to determine how many enrolled infants met criteria for CAPTA notification (even if notification was not yet implemented). Infant data was available for all babies born within the hospital system (*n* = 385). As described in Table 3, six infants were diagnosed with NAS at birth. All had birthing parents in remission for substance use disorders, including three who were on maintenance therapy for opioid use disorder, and two with active Opioid Use Disorder, during the study period. Moreover, five of the six mothers had additional medical complications reported, and two also experienced psychiatric symptoms, including depression.

**Table 3:** Infant characteristics: CAPTA notification and child welfare involvement.

Variable		Infant
CAPTA notification (*n* = 385)	NAS at Birth	1.6%
	FASD at Birth	0.0%
	Positive Toxicology + Withdrawal Symptoms (without FASD or NAS)	0.0%
Child welfare involvement (*n* = 403)	Indicated SCR Report	4.2%
	Foster Care Placements	1.0%

Fifteen infants received a toxicology screen at birth, of which eight were positive (mostly for cannabis alone); no infant who received a toxicology screen was reported to have withdrawal symptoms. Finally, no infants were diagnosed with Fetal Alcohol Spectrum Disorder (FASD) at birth. In total, 1.6% of infants in this sample were eligible for CAPTA notification.

The rate of 16 CAPTA notifications per 1,000 births is much higher than the estimated overall rate at our hospital since CAPTA notification was implemented (four per 1,000 births). These rates are also more than double those reflected by national 2020 Healthcare Cost and Utilization Project data (six NAS diagnoses per 1,000 births). The sample recruited for this study seems to indeed reflect a population at higher risk of CAPTA identifiers (40). Here, the notifications are driven by NAS but not FASD, even though active substance users in this sample were much more likely to meet diagnostic criteria for Alcohol Use Disorder than Opioid Use Disorder. However, 7.5% of participants had a historical Opioid Use Disorder diagnosis, and may have engaged in MAT. A pregnant person receiving MAT under medical supervision may still deliver a child with NAS, and therefore that infant’s birth would be tracked via CAPTA notification, as was the case with 3 of the 6 notifications in this sample.

### 3.6 Infant child welfare involvement

Child welfare data, including the number of substantiated reports for child maltreatment, and the occurrence of foster care placement within the first 6 months of the infant’s life, were collected for all 403 infants (Table 3). Seventeen infants (4.2%) had indicated SCR reports during this period. Furthermore, four infants (1.0%) were placed into care during that 6-month period. In two cases, the infant tested positive for substances (PCP or cannabis) at birth, and in the other two cases, the mother had serious comorbid mental health issues (bipolar disorder and schizoaffective disorder, or schizophrenia). The infant who tested positive for PCP was also diagnosed with NAS at birth, and therefore fell under CAPTA notification requirements. To further contextualize this child welfare data, it may be helpful to consider that in 2023, 1.6% of Bronx children were involved in indicated child welfare investigations, and 0.3% were placed into foster care (41). The rates of indication and placement in this sample are more than twice as high as found in the Bronx overall.

Interestingly, only one of the SCR reports (and the corresponding foster care placement) was for an infant requiring CAPTA notification. This suggests that although CAPTA notification may theoretically overlap with child welfare involvement, these constructs are capturing different issues. For instance, an infant involved in CAPTA notification due to NAS may have a mother taking prescribed medications under the care of a physician, and therefore the infant’s NAS would not be due to parental abuse or neglect. On the other hand, an infant may be born without requiring CAPTA notification, but may be involved with the child welfare system because substance use impacts the parent’s ability to care for the child (for instance, a mother is using cannabis as self-medication to manage a serious psychiatric illness, and that illness – as well as acute cannabis intoxication - may prevent the parent from adequately supervising the infant).

## 4 Conclusion

The CAPTA and CARA regulations have highlighted the problem of newborns prenatally exposed to substances, including opioids, and have required states to consider how to implement these federal requirements. In New York State, although substance use during pregnancy is not explicitly criminalized (as it is in some other states), implementation of CAPTA notifications and Plans of Safe Care (POSCs) are still complex.

### 4.1 Implications

Every single infant who is exposed to, and affected by, prenatal substance use is an infant whose trajectory can be positively impacted by interventions supporting their well-being during the incredibly vulnerable time they are in utero. Steps taken to protect these infants and support the health and well-being of their birthing parents are clinically meaningful and should be applauded. Indeed, when considering this issue from a public health perspective, it is apparent that rates of indicated child maltreatment and foster care placement are more than twice of the rates found throughout the Bronx (41). This may reflect greater vulnerability of infants to child maltreatment, greater risk of abuse or neglect perpetration with complex behavioral concerns (such as substance use and mental health concerns), greater caution of child welfare providers when tasked with ensuring the safety of the youngest of us, or a combination of all three factors. At the same time, these numbers are much smaller than may be expected given the impetus behind this legislation. In particular, the foster care rate of 1% found in this sample is much smaller than found in a sample of infants receiving POSCs in Delaware (11.3%) or North Carolina (4.6%). Finally, 1.6% of infants required CAPTA notification; though much higher in this sample than the overall rates at our hospital and nationally available data (40), it is once again much smaller than might be expected when recruiting such a high-risk sample. Considering societal discussions about the criminalization of motherhood and government intrusion into personal and family affairs (21), the low rates of SCR reports, foster care placements, and CAPTA notification suggest that some of these concerns may be overstated, at least given the clinical details provided by this sample. At the same time, the families involved experienced complex, multifactorial stressors, including substance use and mental health concerns, which impacted their infants.

Another important consideration is the seemingly contradictory finding that substance use rates in this sample is both quite low and quite high. Given the steps taken to recruit a high-risk sample, the low ASI scores, and the perhaps unexpectedly low prevalence of active or in-remission SUD in this sample, may be unexpected. On the other hand, compared to nationally representative data about active SUD rates at delivery (37), the rates of active SUD in this sample are very high. This apparent paradox may be because some participants underreported their use (due to fear, stigma, or mistrust) thereby being less likely to meet diagnostic criteria or screening out of the study altogether. These unidentified parents could have benefitted from Plans of Safe Care and referrals for SUD and other services. Nevertheless, it is important to note that such underreporting would not impact CAPTA reporting, which is based on clinician observations of the neonate. Moreover, the inconsistency between self-report scores and clinically derived diagnostic scores suggests the importance of assessing substance use with universal verbal screening followed by complementary methods (i.e., self-report instruments, clinical interviewing, use of clinical/medical indicators, and judicious use of laboratory testing where indicated). Additionally, assessing at different time points will provide the patient multiple opportunities for disclosure, and patients may be more likely to disclose use over time, once they have built a rapport with the provider.

Finally, we must acknowledge the relatively high rates of psychiatric distress in the birthing parent compared to community and representative samples (38, 39). As Plans of Safe Care are created to address not just substance use concerns, the POSC may be an important avenue to ensure mental health support and trauma resources for pregnant people, especially since a large literature exists on mental health vulnerability during pregnancy and the postpartum period.

### 4.2 Limitations

Novelty of this study includes being able to recruit a large sample, from a large hospital system, which increases generalizability of these data. In addition, this study is timely as all states are working on implementing CARA and CAPTA legislation and it is our hope that our findings will be informative in this process. However, this study has several limitations.

One set of limitations are the low prevalence of active SUD (and low ASI scores) in this sample; despite SUD rates much higher than community samples. While it’s possible this captures the true nature of the underlying population, participant underreporting is also possible. To address this, we provided multiple opportunities to disclose (self report and diagnostic interview) at several time points; the study also incorporated clinical data about toxicology screens from the medical record where possible. Nevertheless, we recognize there may still be a gap between what is reported and the true experience of some participants.

Another set of limitations is inherent to the real-world setting of this research. Although this strengthens the external validity of this work, this also means that participants were enrolled using different methods (verbal screening vs. urine toxicology vs. clinician referral), and that some information (such as information about maternal toxicology screens in the 6-months after enrollment) are based on providers’ clinical decisions rather than a study protocol. We believe our real-world setting strengthens the applicability of this research, but it can also be viewed as a limitation of the study design.

### 4.3 Key findings and recommendations

This study provides data on implementing CAPTA and CARA within the New York City context and reflects some of our clinical findings. Although the rate of active substance use disorders is lower than found in the community, it is still much higher than may be found in other pregnant samples. This is especially concerning given the vulnerable status of infants exposed to substances prenatally and highlights the necessity of services to address the needs of these parents-to-be and their babies. Moreover, the rates of psychiatric issues and trauma were higher than expected in a community sample, and suggests any Plan of Safe Care should integrate psychiatric supports as appropriate.

The rate of CAPTA notifications was higher here than in other samples and NAS was the primary driver. However, it should be noted that half of the infants identified potentially had parents engaged in medication assisted treatment for Opioid Use Disorder. Additionally, instances of adverse child welfare outcomes seem connected to active substance use during pregnancy, as well as serious mental health concerns in the sample. Of the infants who were placed into foster care by ACS, maternal mental health and/or substance use pathology was apparent. However, there were clearly others in this sample who had comorbid substance use, mental health, and trauma symptoms that did not experience adverse child welfare outcomes- suggesting that maternal comorbidity alone is not determinative of child welfare outcomes.

Our recommendations to other hospital systems working to implement CAPTA and CARA legislation are as follows: From a policy perspective, we recommend: (i) Systematic implementation of evidence-based verbal screening related to substance use, to minimize implicit and explicit bias in the process, (ii) Judicious use of urine toxicology screening where clinically indicated, and with consent documented, and (iii) Ensuring that Plans of Safe Care clearly define roles and responsibilities of the involved stakeholders, especially given the interrelationship between substance use and mental health concerns, and the association between these clinical concerns and potential child welfare involvement (24).

## Data Availability

The raw data supporting the conclusions of this article will be made available by the authors, without undue reservation.

## References

[1] Lipari RN Van Horn SL. Children living with parents who have a substance use disorder. Rockville, MD: Center for Behavioral Health Statistics and Quality, Substance Abuse and Mental Health Services Administration (2017). 29144715

[2] Meinhofer A Angleró-DY. Trends in foster care entry among children removed from their homes because of parental drug use, 2000 to 2017. JAMA Pediatr. (2019) 173:881–3. doi: 10.1001/jamapediatrics.2019.1738, PMID: 31305925 PMC6632152

[3] He Y Leventhal JM Gaither JR Jones EA Kistin CJ. Trends from 2005 to 2018 in child maltreatment outcomes with caregivers’ substance use. Child Abuse Negl. (2022) 131:131. doi: 10.1016/j.chiabu.2022.105781, PMID: 35820322

[4] Havens JR Simmons LA Shannon LM Hansen WF. Factors associated with substance use during pregnancy: results from a national sample. Drug Alcohol Depend. (2009) 99:89–95. doi: 10.1016/j.drugalcdep.2008.07.010, PMID: 18778900

[5] The National Survey on Drug use and Health. (2018). Substance abuse and mental health services administration website. Available online at: . (https://www.samhsa.gov/data/sites/default/files/cbhsq-reports/Assistant-Secretary-nsduh2018_presentation.pdf).

[6] NIDA. (2021). National institute on drug abuse website. Available online at: . (https://nida.nih.gov/publications/research-reports/substance-use-in-women/summary).

[7] Behnke M Smith VC. Prenatal substance abuse: short- and long-term effects on the exposed fetus. Pediatrics. (2013) 131:e1009–24. doi: 10.1542/peds.2012-3931, PMID: 23439891 PMC8194464

[8] Chasnoff IJ McGourty RF Bailey GW. The 4P’s plus screen for substance use in pregnancy: clinical applications and outcomes. J Perinatol. (2005) 25:368–74. doi: 10.1038/sj.jp.7211266, PMID: 15703775

[9] Lloyd MH Akin BA Brook J Chasnoff IJ. The policy to practice gap: factors associated with practitioner knowledge of CAPTA 2010 mandates for identifying and intervening in cases of prenatal alcohol and drug exposure. Fam Soc. (2018) 99:232–43. doi: 10.1177/1044389418785326

[10] Lloyd MH Luczak S Lew S. Planning for safe care or widening the net? A review and analysis of 51 states’ CAPTA policies addressing substance-exposed infants. Child Youth Serv Rev. (2019) 99:343–54. doi: 10.1016/j.childyouth.2019.01.042

[11] Child Welfare Information Gateway. (2019). Children’s bureau website. Available online at: . (https://www.childwelfare.gov/topics/systemwide/laws-policies/statutes/safecare/).

[12] Deutsch SA Donahue J Parker T Hossain J Loiselle C De Jong AR. Impact of plans of safe care on prenatally substance exposed infants. Pediatrics. (2022) 241:54–61.e7. doi: 10.1016/j.jpeds.2021.10.032, PMID: 34699908 PMC8792271

[13] Austin AE Shanahan ME Rosemond P Berkoff MC Joyner C Proescholdbell S. Implementation of the North Carolina plan of safe care in Wake County, North Carolina. N C Med J. (2022) 83:67–74. doi: 10.18043/ncm.83.1.67, PMID: 34980658 PMC12925365

[14] Kremer ME Arora KS. Clinical, ethical, and legal considerations in pregnant women with opioid abuse. Obstet Gynecol. (2015) 126:474–8. doi: 10.1097/AOG.0000000000000991, PMID: 26244538

[15] Substance use during pregnancy. (2022). Guttmacher institute website. Available online at: . (https://www.guttmacher.org/state-policy/explore/substance-use-during-pregnancy).

[16] How states handle drug use during pregnancy. (2022). ProPublica website. Available online at: . (https://projects.propublica.org/graphics/maternity-drug-policies-by-state).

[17] Substance use during pregnancy. (2022). American college of obstetricians and gynecologists website. Available online at: . (https://www.acog.org/advocacy/policy-priorities/substance-use-disorder-in-pregnancy).

[18] Patrick SW Schiff DM. A public health response to opioid use in pregnancy. Pediatrics. (2017) 139:e20164070. doi: 10.1542/peds.2016-4070, PMID: 28219965

[19] Olaniyan A Hawk M Mendez DD Albert SM Jarlenski M Chang JC. Racial inequities in drug tests ordered by clinicians for pregnant people who disclose prenatal substance use. Obstet Gynecol. (2023) 142:1169–78. doi: 10.1097/AOG.0000000000005385, PMID: 37769307

[20] Schoneich S Plegue M Waidley V McCabe K Wu J Chandanabhumma PP. Incidence of newborn drug testing and variations by birthing parent race and ethnicity before and after recreational cannabis legalization. JAMA Netw Open. (2023) 6:e232058. doi: 10.1001/jamanetworkopen.2023.2058, PMID: 36884249 PMC9996400

[21] Perritt J. #WhiteCoatsForBlackLives – addressing physicians’ complicity in criminalizing communities. N Engl J Med. (2020) 383:1804–6. doi: 10.1056/NEJMp2023305, PMID: 33211926

[22] Tucker EB. Mandated reporting of perinatal substance use the root of inequity. JAMA Pediatr. (2022) 176:1073–5. doi: 10.1001/jamapediatrics.2022.3404, PMID: 36121662

[23] Merritt D. Lived experiences of racism among child welfare-involved parents. Race Soc Probl. (2021) 13:63–72. doi: 10.1007/s12552-021-09316-5

[24] Madora M Wetzler S Jose A Bernstein PS. Pregnant and postpartum people with substance use disorders: understanding the obstetrical care provider’s roles and responsibilities. Matern Child Health J. (2022) 26:1409–14. doi: 10.1007/s10995-022-03446-x, PMID: 35596847

[25] Department of Health. (2023). Bronx county health indicators by race and ethnicity, 2019-2021. Available online at: . (https://www.health.ny.gov/statistics/community/minority/county/bronx.htm).

[26] Department of Health. (2023). Bronx county hospitals maternity information. Available online at: . (https://www.health.ny.gov/statistics/facilities/hospital/maternity/bronx.htm).

[27] Yonkers KA Gotman N Kershaw T Forray A Howell HB Rounsaville BJ. Screening for prenatal substance use - development of the substance use risk profile – pregnancy scale. Obstet Gynecol. (2010) 116:827–33. doi: 10.1097/AOG.0b013e3181ed8290, PMID: 20859145 PMC3103106

[28] McLellan AT Kushner H Metzger D. The fifth edition of the addiction severity index. J Subst Abus Treat. (1992) 9:199–213. doi: 10.1016/0740-5472(92)90062-S 1334156

[29] D’Angelo A Henke J Bess R. (2019). Regional partnership grants cross-site design report. Mathematica policy research. Available online at: . (https://rpg-cse.acf.hhs.gov/sites/default/files/2024-03/RPG4DesignReport.pdf).

[30] Beck AT Epstein N Brown G Steer R. An inventory for measuring clinical anxiety: psychometric properties. J Consult Clin Psychol. (1988) 56:893–7. doi: 10.1037/0022-006X.56.6.893, PMID: 3204199

[31] Creamer FJ Bell R. The Beck anxiety inventory in a non-clinical sample. Behav Res Ther. (1995) 33:477–85. doi: 10.1016/0005-7967(94)00082-U, PMID: 7755538

[32] Radloff LS. The CES-D scale: a self-report depression scale for research in the general population. Appl Psychol Meas. (1977) 1:385–401. doi: 10.1177/014662167700100306

[33] Elliott DM Briere JN. Sexual abuse trauma among professional women: validating the trauma symptoms Checklist-40 (TSC-40). Child Abuse Negl. (1992) 16:391–8. doi: 10.1016/0145-2134(92)90048-V, PMID: 1617473

[34] Department of Health. (2023). NYS CAPTA CARA information and resources. Available online at: . (https://www.health.ny.gov/prevention/captacara/#what_is_a_plan_of_safe_care).

[35] Statistical Analysis Software. Users’ Guide Statistics Version 9.4. Cary: SAS Institute Inc. (2013).

[36] US Census Bureau. (2024). QuickFacts Bronx County, NY website. Available online at: . (https://www.census.gov/quickfacts/fact/table/bronxcountynewyork/PST045222).

[37] Jarlensky M Krans EE. Co-occurring substance use disorders identified among delivery hospitalizations in the United States. J Addict Med. (2021) 15:504–7. doi: 10.1097/ADM.0000000000000792, PMID: 33273252 PMC8166954

[38] Howard LM Kalifeh H. Perinatal mental health: a review of progress and challenges. World Psychiatry. (2020) 19:313–27. doi: 10.1002/wps.20769, PMID: 32931106 PMC7491613

[39] Yildiz PD Ayers S Phillips L. The prevalence of posttraumatic stress disorder in pregnancy and after birth: a systematic review and meta-analysis. J Affect Disord. (2017) 208:634–45. doi: 10.1016/j.jad.2016.10.009, PMID: 27865585

[40] Centers for disease control and prevention. (2024). Data and statistics about opioid use during pregnancy. Available online at: . (https://www.cdc.gov/pregnancy/opioids/data.html).

[41] NYC Administration for Children’s Services. (2024). 2023 child welfare, Juvenile justice and child care voucher snapshot. Available online at: . (https://www.nyc.gov/assets/acs/pdf/data-analysis/2023/cd-snapshot-bx-1.pdf).

